# Surgical Treatment of Neuroendocrine Liver Metastases

**DOI:** 10.1155/2012/146590

**Published:** 2012-01-26

**Authors:** Ser Yee Lee, Peng Chung Cheow, Jin Yao Teo, London L. P. J. Ooi

**Affiliations:** ^1^Department of Surgical Oncology, National Cancer Centre, 11 Hospital Drive, Singapore 169610; ^2^Department of General Surgery, Singapore General Hospital, Outram Road, Singapore 169608; ^3^Duke-NUS Graduate Medical School, 8 College Road, Singapore 169857

## Abstract

Management of Neuroendocrine liver metastases (NELM) is challenging. The presence of NELM worsens survival outcome and almost 10% of all liver metastases are neuroendocrine in origin. There is no firm consensus on the optimal treatment strategy for NELM. A systematic search of the PubMed database was performed from 1995–2010, to collate the current evidence and formulate a sound management algorithm. There are 22 case series with a total of 793 patients who had undergone surgery for NELM. The overall survival ranges from 46–86% at 5 years, 35–79% at 10 years, and the median survival ranges from 52–123 months. After successful cytoreductive surgery, the mean duration of symptom reduction is between 16–26 months, and the 5-year recurrence/progression rate ranges from 59–76%. Five studies evaluated the efficacy of a combination cytoreductive strategy reporting survival rate of ranging from 83% at 3 years to 50% at 10 years. To date, there is no level 1 evidence comparing surgery versus other liver-directed treatment options for NELM. An aggressive surgical approach, including combination with additional liver-directed procedures is recommended as it leads to long-term survival, significant long-term palliation, and a good quality of life. A multidisciplinary approach should be established as the platform for decision making.

## 1. Introduction

Neuroendocrine tumors (NETs) are a varied group of neoplasms characterized by a relatively slow growth rate and the potential to produce and secrete a variety of hormones along with other vasoactive substances, giving rise to a variety of clinical syndromes. Neuroendocrine tumors are relatively uncommon with an approximate incidence of 1 to 5 per 100,000, but there has been a slow but steady rise in its incidence and prevalence [1, 2]. In the USA, the Surveillance, Epidemiology, and End Results (SEER) database showed a significant increase in reported incidence from about 1 in 100,000 in 1973 to 5 in 100,000 in 2004 [3]. Overall, the incidence is increasing at a rate of 3% to 10% per year [4]. This increase was likely caused in part by improvements in classification of these tumors, and the widespread use of endoscopy for cancer screening likely also contributed to the increase in reported incidence of gastrointestinal NETs [3]. 

Neuroendocrine tumors include carcinoid tumours, gastrinomas, insulinomas, glucagonomas, somatostatinomas, and vipomas [5]. Histopathologically, NETs are tumours of cells, which originate from the neuroectoderm and possess secretory granules. They can occur as part of multiple endocrine neoplasia type 1 (MEN type I) syndrome, or more often they occur in isolation. Anatomically, they are classified according to their site of origin as foregut (including lung), midgut, or hindgut tumours. Clinically, they can be classified as either functional tumors or as nonfunctional tumors [6, 7]. 

The natural history of NETs is highly variable, and clinical management is challenging. Over the past three decades, the understanding of neuroendocrine tumors has been improved significantly by the elucidation of its tumor biology, advances in surgical and perioperative care, and the development of novel diagnostic methods, but the survival of patients with NETs has not improved appreciably in either the USA or UK [1, 8]. 

About 85% of NETs originate from the gastrointestinal tract, and the majority of patients present at diagnosis with metastases. Liver is the most common organ involved, followed by bone and lung [9, 10]. Almost 10% of all liver metastases are neuroendocrine in origin [11–14]. Neuroendocrine liver metastases (NELM) occur in 50% to 75% of small-bowel carcinoids, 5% to 70% of foregut carcinoids, and about 14% of hindgut carcinoids [1, 10]. Up to 85% of NETs have hepatic metastasis (Up to 87% present as synchronous lesions; about 10% as metachronous lesions), and they are potentially completely resectable in only 7% to 15% of patients [10, 15]. 

The presence of neuroendocrine liver metastases worsens survival outcome. There are many treatment strategies that have been attempted over the years for metastatic NETs. These include surgery, locoregional directed therapies such as radiofrequency ablation (RFA), hepatic artery embolization, and transarterial chemoembolization (TACE). These are often used in combination with other systemic therapy such as somatostatin analogues, various chemotherapy regimes, and most recently, peptide receptor radionuclide therapy (PRRT). 

In this paper, we review the current literature and discuss on the surgical aspects of the management of neuroendocrine liver metastases. The spectrum of hepatic surgical procedures comprised complete resections of various extent (including hilar lymphadenectomy), palliative cytoreductive resection, and orthotopic liver transplantation (OLT). The aim of this paper is to collate the available and current information on the management of neuroendocrine liver metastases, to formulate a sound management algorithm, and to promote discussion regarding the role of surgical resection for NELM in these patients.

## 2. Surgery for Neuroendocrine Liver Metastases

There are more reports of patients undergoing surgery for NELM over the past decade than ever before; this is a testament to the increasing acceptance of the aggressive surgical attitude and its associated benefits towards management of NELM [16]. More than 2 decades ago, the Mayo Clinic, in their review of all their patients with carcinoid tumors treated between 1970 and 1989, fewer than 10% underwent cytoreductive hepatic surgery for metastases (*n* = 37) [17]. In 1986, Galland and Blumgart found only two candidates for suitable hepatic surgery among 30 patients with NETs [18]. Sarmiento et al. in a 2002 review paper on NELM, performed a literature search of the English language medical literature from 1973 to 1999, and this revealed a total of only 57 patients who undergone partial hepatectomy for NELM. This is in contrast to another more recent study from the Mayo Clinic group, they reported 170 patients who undergone surgical resection for NELM from 1977 to 1998 [19]. Another recent study comparing liver resection versus intra-arterial therapy for NELM involving nine institutions, they reported more than 300 patients who undergone surgery for NELM [20]. 

The gold standard for evidence-based clinical practice remains unbiased large prospective randomised controlled trials. A multi-institutional collaborative trial conducted over many years may be necessary to recruit patients to compare surgery versus other liver-directed treatment options for NELM, for example, RFA, TACE, and OLT, a study like this will require a minimum of 776 patients in order for it to be powered to detect a difference of 10% in survival rate (based on alpha of 0.05 and power of 0.8) [21]. In view of the highly selective nature of patients undergoing either hepatic resection or OLT, randomized controlled trials evaluating patient outcomes with these treatment modalities would likely be difficult to perform. However, it is important to highlight that due to the paucity of cases even in large institutions and the nonrandomized uncontrolled nature of these studies, there will be biases; the encouraging survival outcomes observed in surgically treated patients may be related to inherently favorable prognostic factors such as low tumor burden.

In the literature, many define curative resection differently. In some studies, curative intent is defined only with a *R*_0_ resection achieved, whereas others classify resection as complete without referring to the margin status, and others defined a curative procedure when all visible gross disease was removed [22]. Despite the armamentarium of treatment options and an ill-defined optimal management protocol, surgical resection of neuroendocrine liver metastases (NELM), if achievable, is generally considered as the best option of both cure and palliation of symptoms [23].

## 3. Curative Liver Resection for Neuroendocrine Liver Metastases

In the principles of surgical oncology, curative resection is defined as the complete removal of tumour tissues with a clear resection margin on pathological examination (hepatic and extrahepatic *R*_0_ status). Liver resection for metastatic disease has gained wide acceptance as a potentially curative option in patients with colorectal cancer [19, 24, 25]. With improvements in the safety of major hepatic resection and an operative mortality rate of about 5% in most series, there has been an increasing role of liver resection for a potential cure of metastatic disease from neuroendocrine tumors.

### 3.1. Results of Curative Liver Resection for Neuroendocrine Liver Metastases

Surgery for NELM is the standard against all other forms of liver-directed or systemic therapies. Due to the relative low incidence, the biological heterogeneity NETs, and NELM, there is a lack of prospective randomized studies providing level 1 evidence. Based on encouraging results from large retrospective studies and cumulative experience, radical surgery including resection of the primary tumour and the liver metastases has been the main treatment for potentially resectable advanced neuroendocrine tumours metastatic to the liver. 

Patients with untreated hepatic metastases have a 5-year survival of approximately 20% to 40% [23, 26]. As a result, many studies have advocated aggressive surgery for NELM with the aim of extending survival (Tables 1 and 2) [19, 23, 26–33]. Due to the indolent nature of the disease, the overall survival is still very good after hepatic resection. This holds true even in stage-4 disease and notwithstanding a high post-curative resection 5-year recurrence rate of more than 40–70% in most series. The overall survival ranges from 46 to 86 percent at 5 years and 35 to 79 percent at 10 years [19, 23, 26, 29, 30, 32, 34–36]. The major studies of liver resection for NELM are presented and summarized in Table 1. Patients in whom hepatic resection was achievable had a significantly better median overall survival and 5-year survival than those with unresectable hepatic disease [26, 30, 31, 37]. The median survival ranges from 52 to 123 months for patients who undergone resection of NELM [20, 38]. 

In the major series attempting curative resection for NELM, curative resection is achieved in a range of 22% to 84% (Table 1) [16, 30]. Sarmiento at al. reported one of the largest single center series on resection for NELM, they achieved complete resection in 44% of their patients. The main site of residual disease resulting in incomplete resection was the liver (96%). They reported a morbidity of 14% and an operative mortality of 1.2%. Not surprisingly, there is a significant difference in recurrence rate in patients with complete and incomplete resection (76% versus 91% at 5 years; *P* = 0.0004) The overall survival rate for the cohort of 170 patients at 5 and 10 years were 61% and 35%, respectively and median survival was 81 months. Notably, they did not detect a difference in survival rate in patient with or without preoperative endocrinopathy, although there was no mention of any quality of life measures [19]. 

Mayo et al. reported the largest and the only multi-institutional experience of surgical management of NELM. In this study of 339 patients, the majority underwent resection of the NELM (77.6%), 19.5% underwent resection plus ablation, and in 2.9%, ablation was the only liver-directed therapy performed. They achieved curative resection (*R*_0_  status) in 53.7% of the patients. In their multivariate analysis, they found in those patients NET without hormonal function, presence of synchronous disease, and concomitant extrahepatic disease as negative prognostic factors. Patients with a hormonally functional NET who had *R*_0_/*R*_1_ resection benefited the most from surgery. In this study, they achieved an overall 5- and 10-year survival of 74 and 51% [39].

The differences in survival data must be interpreted appropriately as the criteria of resectability are ever evolving and there are major improvements in the safety of liver resection, this must be taken into account when comparing recent series to older studies [40]. 

It is important to highlight that even with an aggressive policy and available expertise, conventional partial hepatectomy is rarely possible, as approximately 90% of metastases are multifocal and bilobar [6]. Even in the scenario when complete resection of NLM is performed, early recurrence is more common compared to other common hepatic lesions such as colorectal metastases [40, 41]. 

The biological behaviour of NETs and their metastases is variable, patients with NELM from bronchopulmonary endocrine tumors are known to have the poorest prognoses as compared to other sites [42, 43]. Patients with NELM from a colonic primary seem to have a better recurrence free survival when compared to the rest of the other sites [42]. Other than tumor site, independent preoperative factors for a poor prognosis include tumor differentiation, pancreatic tumor, nonfunctional primary tumor, presence of multiple and/or bilobar liver metastases, and invasion of greater than 75% of the hepatic parenchyma [23, 29, 44]. 

There are studies comparing resection against other forms of therapy in an attempt to identify a subset of patients who will benefit the most from surgery and to individualize therapy regimes [45]. In a recent multi-institutional study analyzing over 700 patients comparing liver resection versus intra-arterial therapy (including transarterial chemoembolization, bland transarterial embolization, and drug-eluting beads or yttrium-90) for NELM, they found that although surgical management provided a survival benefit over intra-arterial therapy among symptomatic patients with >25% liver involvement, there was no significant difference in long-term outcome. In this study, liver-directed surgery includes resection and RFA or in combination. They concluded that asymptomatic patients with a large burden of liver disease benefited the least from surgery and nonsurgical liver-directed therapy like various forms of intra-arterial therapy may be more appropriate. They suggested that surgical resection of NELM should be reserved for patients with low-volume disease or for those patients with symptomatic high-volume disease [20]. 

Although there is no significant difference in disease incidence worldwide, most of the studies on NELM are from the West. There is only a single report from Asia reporting a case series of 21 patients reporting a similar results and conclusions. They report an overall 68% 3-year and a 41% 5-year survival rate for patients; those that achieved curative resection have a significantly better 5-year survival rate (73% versus 0%, *P* = 0.01) compared to those who underwent a palliative resection [43]. The group from M.D. Anderson Cancer Center reported recently in their study of 172 patients undergoing surgery for NELM that they found that in Asians, on multivariate analysis, the recurrence free survival was significantly lower as compared to the rest of the study population [42]. 

There is no evidence for the effect of adjuvant treatment of radically operated patients [46]. Medical treatment is required only in the event that the tumour and/or its metastases cannot be completely resected.

## 4. Palliative Liver Resection for NET Liver Metastases

A distinguishing feature of these malignancies beside the ability to metastasize is the potential for unregulated endocrine activity. This fact complicates treatment but serves as one of the main rationale for roles of palliative surgery. Even when resection with curative intent is not feasible, either due to the presence of extrahepatic disease or extensive intrahepatic disease, there remains a role for surgery, though this remains less well defined. The goals of palliative surgery focus on retardation of tumor cell growth, relief of mass symptoms, symptoms of hormonal hyper-secretion, prolonging survival, and finally achieving a good quality of life on the long term.

Some authors have defined cytoreductive hepatic surgery as resection of 90% of the bulk of the tumour, and this refers to the incomplete resection of tumor to reduce symptoms and facilitate the effect of nonsurgical strategies [47]. DebuIking surgery is defined as surgical resection with gross residual disease beyond the criteria of cytoreductive surgery. The Mayo Clinic group has recommended that palliative resection is justified if at least 90% of hepatic metastases are resectable, and the extrahepatic tumour bulk is limited [48]. This recommendation has also been advocated separately in the consensus report of the European Neuroendocrine Tumour Society [49]. Frilling et al. proposed that cytoreductive liver resections should be considered if there is no evidence of unresectable extrahepatic disease and less than 70 percent of the liver is involved by tumour [22]. 

In the palliative setting, where symptomatic control for quality of life and not extension of the survival is the primary goal, the risk-benefit ratio needs to be clearly ascertained in order to justify surgery as liver resection is not without its share of significant morbidity and mortality. If cytoreductive surgery can increase survival, then the application of operative interventions is doubly justified in a patient population that can survive many years with symptomatic disease. 

Neuroendocrine tumor behavior and biologic characteristics exist to justify the application of cytoreductive therapy. In the majority of NETs, the tumors have a long doubling time, especially in gastrointestinal neuroendocrine tumors where hepatic and regional nodal metastases are the predominant site of spread. In the majority of metastatic NETs, metastases are limited to the liver. They are susceptible to chemotherapy and embolization, and the tumor volume correlates with magnitude of disabling endocrine symptoms. Crucially, the primary tumors are often resectable despite extensive metastases [50]. The survival of patients with gastrointestinal neuroendocrine tumors even with established metastases is longer compared with that of patients with other malignancies, up to 30 to 40% 5-year survival without treatment. Metastatic patients with gastrointestinal neuroendocrine tumors clearly have a better survival compared to patients with metastatic adenocarcinoma of the gastrointestinal tract [51, 52].

### 4.1. Results of Palliative Liver Resection for NET Liver Metastases

Proponents of aggressive approaches advocating partial hepatectomy, cytoreductive liver resection, and ablation cite numerous many retrospective institutional studies reporting palliation of symptoms and prolonged survival duration among patients undergoing surgery with curative or near curative intent (Table 1) [13, 17, 26, 53, 54]. 

However, to date, there have been no trials evaluating these criteria in a prospective fashion. Furthermore, these guidelines are neither universally accepted nor adopted, and the majority of the literature consists of heterogeneous cohorts, making interpretation and comparison of data difficult.

Advanced unresectable neuroendocrine tumours are associated with prolonged survival. Fifty percent of incurable tumors survive 5 or more years after diagnosis. Median survival of patients with unresectable hepatic metastases ranges from 3 to 4 years, and nearly 30–40% of these patients were alive at 5 years [52, 55].

Patients may suffer for prolonged periods from metastases-associated endocrinopathies, of which the severity of the endocrinopathy parallels the tumor volume [48]. Although there is an absence of a validated universal method for measuring and comparing symptoms and their response to therapy, the literature suggests that resection of neuroendocrine hepatic liver metastases can result in excellent palliation of hormone-related symptoms. In carefully selected patients, partial or complete relief of systematic endocrine-related symptoms can be achieved in more than 90% of subjects undergoing cytoreductive or palliative liver resection, with a correspondingly low morbidity and mortality [13, 19, 54]. Objectively, one study demonstrated a postoperative reduction in urinary excretion of the serotonin metabolite 5-hydroxyendoleacetic acid (5-HIAA) which correlated with the decrease in hormone-related symptoms of flushing and diarrhea [19]. As such, surgery for symptoms palliation alone is well justified.

After successful cytoreductive surgery, the mean duration of symptom reduction is between 16 to 26 months [13, 56, 57]. Unfortunately, recurrence or progression of symptoms can occur within 20–45 months, and the five year recurrence/progression rate ranges from 59 to 76% [19, 57]. 

More than a decade ago, Que et al. showed that initial symptomatic response is similar in patients with metastatic disease whether resected with curative or palliative intent, but recurrence of symptoms occurred earlier in patients undergoing resection with palliative intent (11.3 months versus 20.4 months). He further proposed that the duration of response can be predicted by the completeness of resection and normalisation of hormonal markers in the immediate postoperative period [13]. Hibi et al. reported the only Asian series of liver resections for metastatic neuroendocrine tumours. Seven patients (33% of the cohort) underwent palliative resection. The resolution of symptoms after surgery was complete in five patients, partial in one, and one was asymptomatic preoperatively [43]. 

Sarmiento et al. have one of the largest single-institution series of hepatic resections for metastatic neuroendocrine tumours with 170 patients. This study included patients with curative resections, but majority of the patients underwent palliative resection (56%). They showed good response in 96% of patients with hormone-related symptoms, and all these patients had at least 90% of gross hepatic disease resected. In their series, symptom recurrence was 59% at 5 years, with a median time to recurrence of 45.5 months, although these were appreciably less severe and easily controlled with minimal doses of octreotide. They reiterated the recommendation that aggressive resection for palliation be pursued with a view to remove at least 90% of gross disease and suggested that the increasing availability and applicability of radiofrequency ablation would further increase the pool of patients who could potentially benefit from a combined ablative policy [19].

Notwithstanding the fact that metastatic neuroendocrine disease confined to the liver is compatible with prolonged survival, pain or debility due to hepatomegaly and symptoms from a variety of endocrinopathies from excessive hormone production can impact negatively on patients' quality of life [51, 58]. There is a paucity of data looking specifically at improvement in quality of life after surgery for NELM. One study by Knox et al. did demonstrate an improvement in quality of life as measured by Karnofsky performance score by the third postoperative month which was sustained for more than 4 years after surgery [59]. 

Radiofrequency ablation (RFA), transarterial chemoembolisation (TACE), cryosurgery, and other ablative therapies have been used for palliation, although a Cochrane review in 2009 found no prospective randomized trials comparing the results of surgery to those of any other method in the palliation of metastatic neuroendocrine tumours to the liver [21]. Bergen and Osborne compared resection outcomes versus embolic treatment in symptomatic metastatic NET and reported that cytoreductive surgery for metastatic neuroendocrine tumors resulted in improved symptomatic relief in terms of high proportion of complete (69% versus 59%) as well as a longer duration of relief. There is also improved survival when compared with embolic therapy in this nonrandomized retrospective study. They recommended cytoreductive surgery should be pursued whenever possible even if complete resection may not be achievable [60].

## 5. Liver Transplantation for Neuroendocrine Liver Metastases

Liver transplantation has a selected role in unresectable NELM and is proposed for certain candidates with a 5-year overall survival of up to 70% and 5-year recurrence free survival of up to 50% [9]. In the largest meta-analysis of 103 patients, the 5-year survival rate was 47%, with only 24% of patients free of disease recurrence [61]. The largest series of liver transplants for NELM was the multicenter French study, coordinated by Le Treut et al. which reported on 85 cases with an overall survival of 47% and a recurrence free survival of 20% at 5 years [62]. 

**Figure 1:**
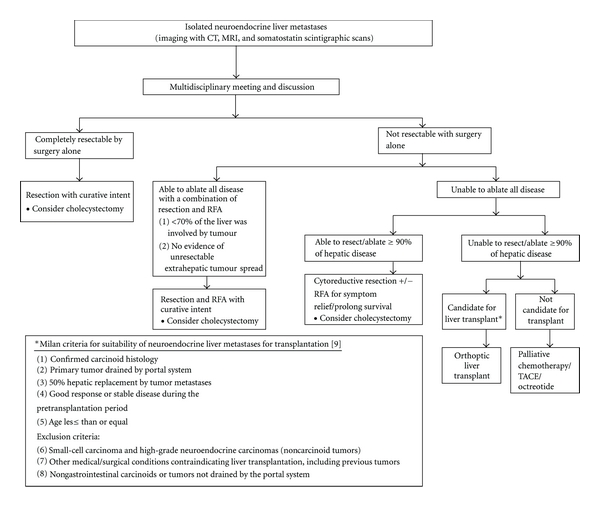
Proposed Algorithm for management of isolated neuroendocrine liver metastases.

Mazzaferro et al. proposed a set of guidelines for the selection of candidates for liver transplantation, now known as the “Milan criteria” (Figure 1 inset) [9]. These guidelines emphasized the requirement for a specific diagnosis of endocrine tumors and considered patients who had well-differentiated endocrine tumors with low-grade malignancy, established on the basis of mitotic and proliferation indices as eligible candidates for liver transplantation. In another consensus guideline by the European Neuroendocrine Tumor Society, patients with diffuse unresectable liver metastases or those with life-threatening hormonal disturbances refractory to medical therapy, liver transplantation may be a possible treatment option for these selected patients. Due to the slow-growing nature of NETs and their tendency to metastasize only to the liver, NETs remain one of the few indications for liver transplantation in metastatic disease, particularly if living-related donation is feasible. They commented that patients who are most likely to benefit from liver transplantation are those less than 50 years old who are free of extrahepatic tumor and have low expression of Ki-67 and E-cadherin [63]. The details and results of OLT for NELM are beyond the scope of this paper but in brief, several factors plague liver transplantation as an effective treatment option. Although liver transplantation has the theoretical advantage of removing all tumor burden in patients beyond the criteria for respectability, and advances in technique and improved perioperative care have made liver transplantation safer recently; early disease recurrence, significant morbidity and mortality, the absence of extensive experience, and shortage of donor organs all contribute to preclude orthotopic liver transplantation (OLT) as an effective option for most patients with unresectable NELM [64].

## 6. Combination Therapy

Palliation can also be achieved with a combination of treatment modalities. Surgery can be combined with other therapies, for example, antihormonal therapy, chemotherapy, immunotherapy, and interventional radiological procedures, either simultaneous or in stages. The role of adjuvant therapy and which modality will be optimal in complementing palliative surgery is not well established. 

There is some data on the efficacy of a cytoreductive strategy combining both surgery and RFA in the same session (Table 2). Eriksson et al. showed that out of the patients aggressively treated with a combination of anatomical and nonanatomical resection with intraoperative and percutaneous radiofrequency ablation, 70.6% of those with carcinoid syndrome had partial or complete symptom response. They also found large tumor size, high preoperative Chromogranin A and 5-HIAA levels, and high Ki67 index to be risk factors for recurrence [65]. Musunuru et al. showed that compared to either medical therapy alone, systemic chemotherapy, or transarterial embolization, a combination of surgery and RFA resulted in 100% symptom control and improved 3-year survival rate (83% versus 31%) [31]. 

## 7. Role of Prophylactic Cholecystectomy

The role of prophylactic cholecystectomy has been recommended by some authors when surgery is considered for an advanced neuroendocrine tumor [49]. The rationale for prophylactic cholecystectomy during partial hepatectomy for NELM includes (1) the fact that somatostatin analogs may induce gallstone disease in up to 50% of cases; (2) chemoembolization has a high risk for cholecystitis occurrence; (3) in the event that the hepatic lesions are not resectable and should the hepatic artery be ligated or embolized to control symptoms, necrosis of the gallbladder can potentially occur. Some authors cite these reasons to justify the minimal morbidity from the additional procedure [53, 66]. However, there are differing opinions, in the recent 2010 Nordic Guidelines, they commented that as the somatostatin-induced bile stones usually are asymptomatic, liver embolization techniques have improved considerably and the risk of complications to cholecystectomy is up to 3%; taking these points into consideration, they did not recommended prophylactic cholecystectomy.

## 8. Surgical Considerations

As neuroendocrine hepatic metastases are numerous and often large, surgical resectability is a primary concern. Once it has been established that curative or cytoreductive resection is indicated, resectability can be determined from two factors: anatomical feasibility and volumetric tolerance. A multidisciplinary approach involving in the minimum a liver surgeon and a dedicated hepatobiliary radiologist, a medical oncologist is recommended to validate the decision.

### 8.1. Resectability of Neuroendocrine Liver Metastases

The definition of “resectability” is determined by many factors including patient, disease, and technical factors. The principle lies in the technical ability to leave a remnant with adequate function for sustaining life but consistent with low perioperative morbidity and mortality and acceptable long-term outcome in survival and quality of life. Relative contraindications to partial hepatectomy include significant medical comorbidities, rapidly progressing intrahepatic disease, and progressive or extensive extrahepatic disease. The golden rule for partial hepatectomy is to ensure enough liver parenchyma with a satisfactory blood supply (hepatic artery, portal vein, and hepatic vein), and biliary drainage remains after resection so that the patient does develop postoperative liver failure. Contraindications of liver metastasectomy are situations in which the tumors have invaded the biliary confluence or invaded the three hepatic veins or the portal bifurcation. Any scenario in which bilioenteric anastomosis to the remaining bile ducts cannot be constructed is also considered as a relative contraindication to resection. Fortunately, the growth pattern of NELM permits an aggressive surgical approach as the lesions are often discrete and the masses displace rather than invade or encase the major intrahepatic vessels or bile ducts. Some NELM have a miliary pattern with or without dominant tumors, however, these miliary metastases does not affect the resectability of the larger dominant tumors, and due to slow growth of the tumors, resection of dominant NELM can be considered for cytoreductive purposes for symptoms relief [50].

The ability of the nonpathological liver to regenerate after liver resection is good, and a remnant functional normal liver tissue as little as 20 to 25% may be sufficient [67, 68]. The ratio of remnant functional liver to the initial total liver tissue may be difficult to estimate because of the high number and size of metastatic nodules; the volume of these tumors should be excluded when determining the volume of the total liver. There are studies that utilize volumetric ratio without considering the total liver volume, but use percentage of total body weight instead [69]. It has been demonstrated that there is a risk of postoperative liver failure when the remaining functional liver ratio falls to less than 0.5% of total body weight. Multidetector computed tomography (CT) of the liver is usually applied to measure liver volumes. Most surgeons will not advocate surgery if the estimated volume of functional remnant liver is either less than 20–25% of the total liver or less than 0.5% of total body weight. Other than Child-Pugh scoring, Model for End-Stage Liver Disease (MELD) and liver function test, some studies have validated the use of indocyanine green clearance (ICG) as an objective adjunct in assessing liver function, in our institution, we use ICG selectively for patient with “borderline respectability” [70–73]. In patients with “borderline respectability”, one safer option is to induce hypertrophy in the remaining functional liver via portal embolization to reduce the risk of postoperative liver failure. Compensatory hypertrophy of the contralateral lobe is generally observed in a period of three to six weeks. Some studies report a high feasibility rate for this strategy and report a mean gain in liver volume of over 40%, thus increasing the volumetric feasibility for resection of hepatic metastases from NETs [74, 75]. In patients whose NELM are bilobar, in line with aggressive approach, studies have reported the two-stage hepatectomy strategy as a useful alternative to portal embolization. This technique enables the successive treatment of the left lobar metastases followed by those in the right lobe, with ligature of the right portal vein in the first stage of the surgery to induce hypertrophy of the left lobe during the time interval between the two operations. In the second stage, a right hepatectomy is performed with the hypertrophied left lobe sustaining postoperative liver function [75]. Some authors suggest a core biopsy should be considered prior consideration for resection, in the situations when the health of the remnant liver is in doubt [42].

### 8.2. Role of Hepatic Lymphadenectomy in Neuroendocrine Liver Metastases

The role of hepatic lymphadenectomy in neuroendocrine liver metastases is not well established [76]. Most of the experience and data are extrapolated from colorectal liver metastasis (CLM), of which nodal involvement of the hepatoduodenal ligament is an independent predictor of survival following a curative partial hepatectomy [77–79]. The Mayo Clinic identified metastatic hepatoduodenal lymph nodes as an independent predictor of survival with an almost 40% increase in 5-year survival (18.8% versus 58.1%) in node-negative patients following hepatectomy for colorectal metastases [77]. A French study on lymph node metastases in CLM reported a superior 3-year survival of 38% in patients with nodal metastases limited to the hepatoduodenal ligament and retropancreatic region, versus no survivors beyond a year in patients with metastases to the common hepatic artery and celiac axis region following a hepatectomy; the authors concluded that a systematic regional lymphadenectomy should be performed in patients undergoing hepatectomy for CLM as it offers prognostic information, however, in the presence of metastases to common hepatic artery and celiac axis region, a hepatectomy may not be justified [80]. These data suggest that a regional lymphadenectomy is important in all patients undergoing a curative hepatectomy for malignant tumors for accurate prognostication, selection of patients for adjuvant therapy, and prospective evaluation of a potential survival benefit. However, there is little information on NELM, and there is no consensus if the CLM experience is translatable to NELM. Criteria for an adequate lymphadenectomy for NELM including the extent and minimum number of lymph nodes removed require further study.

### 8.3. Width of Resection Margins in Neuroendocrine Liver Metastases

Another point of contention in the resection of liver metastases is the optimal margin of resection. There is no clear evidence or consensus on the width of clear margins for NELMs. Generally for liver metastases, a positive resection margin predisposes to marginal recurrences and is an independent predictor of poor survival [81–84]. Most of the experience and data are accumulated from colorectal liver metastasis and extrapolated to other liver metastases from other primaries, for example, NETs. The optimal width of the resection margin is confounded by the different parenchymal transection techniques used at different centers [85]. The loss of a 5- to 8-mm tumor-free margin during liver resection confounds the issue of adequacy of pathologic margins and the use of contrast-enhanced intraoperative ultrasound may enhance the accuracy of resection margins [86, 87]. Comparisons of anatomic and wedge resections for CLM have demonstrated no difference in the rate of positive margins, recurrence patterns, or overall survival [88–90]. Several series demonstrated that a positive resection margin is an independent predictor of poor survival following hepatectomy for CLM, further to this, some centers have demonstrated improved outcomes with more than 1 cm margins compared with narrower margins. The Memorial Sloan Kettering Cancer Center, in their review of 1019 patients after hepatic resection for CLM, reported a significant decrease in the median survival of patients with a positive margins of 1 cm or less, and that a resection margin of more than 1 cm was an independent predictor of survival [91]. In another report by Wakai et al. 95% of the intrahepatic micrometastases were noted within 1 cm of the advancing edge of the metastatic tumor deposit and a margin of 1 cm or more was associated with significantly improved survival [92]. In view of this, some have suggested that nonanatomic resection for CLM should at a minimum attain negative pathologic margins with the goal of a 1 cm margin. In contrast, some authors report that the width of the resection margin does not influence survival as long as it is negative [86, 92–95]. Complete resection is the goal of hepatectomy for neuroendocrine liver metastases as the rate of recurrence and the median time to recurrence are negatively affected by incomplete resection [13, 19, 23]. Agrawal and Belghiti have recently recommended a resection margin of less than 1 cm for noncolorectal liver metastases, that is, neuroendocrine liver metastases [96].

## 9. Perioperative Considerations

There have been huge improvements in surgical and anesthetic techniques and the perioperative management of these patients, these contributed to significant reductions in the morbidity and mortality rates after partial hepatectomy. Even in institutions reporting a significant percentage of complex hepatectomies, perioperative mortality is approaching 1% to 3% in patients without underlying liver dysfunction [97]. Perioperative morbidity and mortality rates for metasectomies of neuroendocrine tumors are similar to those reported for colorectal metastases [13, 98]. The surgery-related mortality of major series of hepatectomy for NELM ranges from 0 to 9% (Table 1) [74]. The reported overall morbidity rate ranged from 3% to 24% after partial hepatectomy for NELM (Table 1). 

Patient selection is important for safe hepatic surgery. Patients with significant comorbidities should be reviewed by the anesthetic team for evaluation of preoperative risk factors related to general health status (American Society of Anesthesiologists (ASA) score) or associated diseases (e.g. right cardiac failure in carcinoid syndrome). Perioperatively or prior to any intervention, for example, radiofrequency ablation, all patients should receive 100–150 *μ*g/h octreotide intravenously for 12 hours prior procedure [22]. Alternatively, preoperative preparation with 150 to 500 *μ*g of somatostatin administered in the preinduction phase in the operating theatre prevents hemodynamic instability intra-operatively. Specific presurgery preparation may be necessary for individual tumors for example, for insulinomas, regular glucose monitoring and for gastrinomas, H_2_-receptor antagonists or H^+^-K^+^ ATPase inhibitors are essential [50]. An endocrinologist consult preoperatively is recommended for patients going for surgery with functional NETs. 

In general, the perioperative risk is not increased with specific endocrinopathies, with the exception of carcinoid heart disease. Surgical repair of carcinoid heart disease may be required prior to hepatic resection for symptomatic carcinoid syndrome in selected patients to reduce the risk of massive hemorrhage caused by intrahepatic venous hypertension from right heart failure [99]. 

Perioperative morbidity and mortality are directly related to the postoperative liver remnant function, the most important determinant of which is the extent of liver resection. In patients with tumors located adjacent to vascular structures or those with multiple lesions exist in separate distinct locations, this group of patients are likely to require large volume resections thus leaving small functional remnant liver as a result. Many strategies have been suggested to cope with this challenge; these include parenchyma-preserving, segmental approaches to resection, incorporation of concomitant wedge excisions or thermal ablations for small tumors outside the perceived safe field of resection, and the use of either preoperative portal vein embolization or staged resections to induce hypertrophy of the future liver remnant [100]. Besides liver-related complications, Glazer et al. also reported severe postoperative complications, for example, intra-abdominal fluid collection as an independent risk factor for perioperative mortality [42].

## 10. Discussion

The presence of liver metastases is a distinguishing feature of malignant neuroendocrine tumors and is the rate-limiting step on patient's survival [101, 102]. Based on available data (Tables 1 and 2), we advocate an aggressive surgical policy and propose an evidence-based surgical management algorithm (Figure 1). Surgery has a strong role in NELM and should be the treatment of choice if patients are fit and disease factors allow for it in both the curative, as well as palliative settings. Strategies to increase the limits of resectability, for example right portal vein embolization to induce hypertrophy of the remaining left lateral section before right hepatectomy or staged hepatic resections, can be considered especially in experienced centres. We propose that a multidisciplinary meeting should be the platform for decision making. In patients with curative lesions, curative resection should be the 1st line treatment, if cure cannot be achieved by surgery alone, ablative modalities, for example, RFA can be combined with surgery to achieve “cure”. Adjuvant systemic therapies and local ablative treatment have a role in complementing surgery for disease control but the exact role of each is not well established and beyond the scope of this paper (Table 2). In the event, if curative intent cannot be achieved, cytoreductive surgery with or without ablation should be performed if at least 90% of the tumour load can be treated. If surgery and ablation cannot achieve this 90%, liver transplantation is a consideration, failing which best medical treatment should be offered for palliation, for example, TACE, Chemotherapy, and octreotide. 

A study from Germany further classified the different patterns of NELM; they defined type I as single metastasis of any size, type II as isolated metastatic bulk accompanied by smaller deposits, with both liver lobes always involved, and type III as disseminated metastatic spread, with both liver lobes always involved with virtually no normal liver parenchyma. With this classification, they found that the 3 types of NELM differ in behaviour and biology and are the only significant independent predictor of survival [22]. This study illustrates that although in general an aggressive therapy is recommended, individualization of treatment strategy should be tailored to each patient as some will benefit more from surgery for NELM than others [20]; a multidisciplinary team approach should be the platform for this decision-making process (Figure 1). The experts participating in such a team can comprise of endocrinologists, gastroenterologists, hepatobiliary surgeons, pathologists, diagnostic and interventional radiologists, medical oncologists, and nuclear medicine physicians. 

Adjuvant therapy is currently not indicated in patients with completely resected NETs, and this need to be further studied in clinical trials [46]. Development of tumor repositories and clinical databases should help provide useful information to facilitate the development of future studies. Analysis of such data should also help identify patient subgroups at particularly high risk of recurrence and to validate scoring systems to help predict those patients most likely to benefit [23]. The development of standardized histopathological and staging criteria should also improve the selection of appropriate patients for clinical studies.

## 11. Conclusions

An aggressive surgical approach leads to long-term survival in patients with NELM. Although long-term cure can only be achieved in a proportion of patients with malignant NETs, significant long-term palliation can be achieved. This aggressive surgical approach can be recommended, keeping in mind that additional liver-directed procedures may be required or combined with surgery to achieve effectiveness for a good quality of life. A multidisciplinary approach in lieu of future prospective, randomized long-term followup studies should be established to identify the group of patients who will most benefit from surgery for NELM.

## Figures and Tables

**Table 1:** Case series of hepatic resections performed for neuroendocrine liver metastases published in the past 15 years. The following table summarizes the results of modern-day series of liver resections performed for neuroendocrine tumors liver metastases.

	Author	Year	*N* undergoing resection	Percentage of curative and palliative resections	Operative mortality (%)	Operative morbidity (%)	Symptom control (%)	Survival (% is for 5-year survival if not otherwise stated)
(1)	Que et al. [13]	1995	74	38% curative 62% palliative	2.7	24	90	73% at 4 years Similar survival rates between curative versus palliative resection
(2)	Dousset et al. [35]	1996	17	71% curative, 29% palliative	5.9	23.5	88	46% at 5 years (overall) 62% at 5 years (curative)
(3)	Ahlman et al. [16]	1996	54	22% curative, 78% palliative	0	NA	100	70% at 5 years
(4)	Chen et al. [26]	1997	15	100% curative	0	NA	NA	73% at 5 years
(5)	Chamberlain et al. [23]	2000	34	44% curative, 56% palliative	6	NA	100	76% at 5 years Differences in survival between curative/palliative resections are not reported
(6)	Grazi et al. [30]	2000	19	84% curative, 16% palliative	0	NA	NA	92.6% at 4 years
(7)	Sarmiento et al. [19]	2001	170	44% curative, 56% palliative	1.2	4.1	96	61% at 5 years; 35% at 10 years Differences in survival between curative and palliative resections are not reported
(8)	Yao et al. [32]	2001	16	100% curative	0	12	NA	70% at 5 years
(9)	Coppa et al. [36]	2001	20	100% curative	NA	NA	NA	67% at 5 years
(10)	Jaeck et al. [103]	2001	13	NA	0	NA	100	68% at 6 years
(11)	Nave et al. [33]	2001	31	32% curative, 68% palliative	0	13	NA	47% at 5 years 86% for curative resections, 26% for palliative resections
(12)	Dejong et al. [104]	2002	5	NA	0	20	NA	Median survival 59 months
(13)	Norton et al. [28]	2003	16	100% curative	0	19	100	82% at 5 years
(14)	Elias et al. [29]	2003	47	53% curative, 47% palliative	5	45	NA	71% at 5 years Similar survival rates between curative versus palliative resection
(15)	Osborne et al. [54]	2006	61	62% curative, 28% palliative	0	3.2	91	80% at 5 years (curative) 60% at 5 years; (palliative)
(16)	Reddy et al. [41]	2006	33	70% curative, 30% palliative	9	42	NA	75% at 3 years; Differences in survival between curative and palliative resections are not reported
(17)	Hibi et al. [43]	2006	21	100% curative	0	19	100	41% at 5 years
(18)	House et al. [37]	2006	26	100% curative	0	NA	NA	Median survival 78 months
(19)	Gomez et al. [34]	2007	18	83% curative, 17% palliative	5.5	22	100	86% at 5 years Differences in survival between curative and palliative resections are not reported
(20)	Landry et al. [105]	2008	23	NA	0	26	NA	75% at 5 years
(21)	Chambers et al. [106]	2008	30	NA	0	22	86	74% at 5 years
(22)	Ahmed et al. [107]	2009	50	NA	0	NA	NA	74.3% at 5 years

Comments: Operative mortality and morbidity refer to figures for patients undergoing liver resections. Survival date is stated for the entire cohort (curative and palliative resections), unless otherwise is stated. Symptom control includes both partial and complete response. NA means that information was not provided in original paper.

**Table 2:** Case series of hepatic resections with ablation performed for neuroendocrine liver metastases.

	Author	Year	*N* undergoing resection/ablation	Operative mortality (%)	Operative morbidity (%)	Symptom control (%)	Survival
(1)	Mayo et al. [39]	2010	339	NA	NA	NA	74% at 5 years
(2)	Glazer et al. [42]	2010	172	0	22.1	NA	77.4% at 5 years 50.4% at 10 years
(3)	Strosberg et al. [108]	2009	31	NA	NA	NA	75% at 5 years; median survival of 103 months
(4)	Touzios et al. [27]	2005	19	5.2	42	95	72% at 5 years
(5)	Musunuru et al. [31]	2006	13	NA	NA	100	83% at 3 years

Some studies [22, 65, 102, 109] are excluded in the tables as the data is not stratified to liver resections and thus no meaningful data can be extracted for liver resections.
